# Simultaneous determination of 137 drugs of abuse, new psychoactive substances, and novel synthetic opioids in meconium by UHPLC-QTOF

**DOI:** 10.1007/s00216-021-03533-y

**Published:** 2021-07-20

**Authors:** Ángela López-Rabuñal, Daniele Di Corcia, Eleonora Amante, Marta Massano, Angelines Cruz-Landeira, Ana de-Castro-Ríos, Alberto Salomone

**Affiliations:** 1 Servizo de Toxicoloxía, Instituto de Ciencias Forenses, Facultade de Medicina, Universidade de Santiago de Compostela, C/ San Francisco s/n, 15782 Santiago de Compostela, Spain; 2 Centro Regionale Antidoping e di Tossicologia, Regione Gonzole 10/1, 10043 Orbassano, Torino Italy; 3 Dipartimento di Chimica, Univesità di Torino, Via Pietro Giuria 5, 10125 Torino, Italy

**Keywords:** Meconium, NPS, QTOF, Fentanyl, In utero drug exposure

## Abstract

New psychoactive substances (NPS) have been introduced into the market in recent years, with new analytes reported every year. The use of these substances in women can occur at any stage of life, even in the childbearing age. Drug use during pregnancy presents significant risks for the mother and the fetus, so it is important to have tools that allow to detect prenatal exposure to these substances of abuse. Therefore, an analytical method for the determination of 137 NPS and other drugs of abuse in meconium by UHPLC-QTOF was developed and validated for semi-quantitative purpose. Linearity range, limit of detection (LOD), precision, matrix effect, selectivity, and specificity were evaluated. For all analytes, the calibration curves were studied in the ranges between 2, 10, or 50 ng/g and 750 or 1000 ng/g, (depending on the analyte) and the LOD ranged between 0.04 and 2.4 ng/g. The method was applied to 30 meconium specimens from cases in which fentanyl had been administered as epidural anesthesia at the time of delivery or cases in which the maternal hair was positive to other drug of abuse. Four meconium samples tested positive for fentanyl (range concentration = 440–750 ng/g) and two samples tested positive to acetylfentanyl (range concentration = 190–1400 ng/g).

## Introduction

New psychoactive substances (NPS) are new chemicals designed to mimic the effects of classic drugs (cocaine, cannabis, heroine, etc.). UNODC was the first to use the term NPS to refer to “substances of abuse, either in a pure form or a preparation, that are not controlled by the 1961 Single Convention on Narcotic Drugs or the 1971 Convention on Psychotropic Substances, but which may pose a public health threat” [1]. Different NPS are reported every year and their presence has already been detected in more than 100 countries. Due to their unregulated status, these drugs were initially sold on the Internet as “legal highs” or “bath salts,” so efforts have been made to speed up the legislation on their production and distribution [2]. The main substance groups of NPS present in the market in 2019 were stimulants (36%), synthetic cannabinoids (31%), classic hallucinogens (15%), and opioids (8%) [1].

To collect information about the prevalence of use, several countries have recently included NPS in their national drug surveys. In 2017, 1% of the Spanish adult population consumed NPS [3] compared to the 2–11% who consumed classical drugs such as cannabis or cocaine [4]. The prevalence of NPS use decreases to 0.7% in the group of women of childbearing age (15–44 years old) [4]. Opioids are the NPS group of greatest concern nowadays. In the USA, the opioid epidemic is caused by the increasing prevalence of the use of synthetic opioids and fentanyl analogs. Although this is not yet the situation in Europe, several concerns have been raised [5, 6]. Moreover, in Spain between 8.3 and 18.3% of women of childbearing age admitted using analgesic opioids at some point in their lives [4].

Pregnant women are a very vulnerable group to the harmful effects of drugs because its use during pregnancy can have negative effects on both the mother and the fetus. Monitoring of opioids use during pregnancy is especially important, since besides their possible illicit use, these drugs are used for pain management (epidural anesthesia) or to treat drug addiction [7]. Prenatal exposure to drugs is mostly related to neonatal abstinence syndrome (NAS), but also to low birth weight and preterm delivery [8–10]. Therefore, it is important to detect prenatal exposure to psychoactive substances. Meconium analysis is considered the gold standard for detection of prenatal drug exposure [11, 12]. Meconium is the first stool of newborn and its composition is very complex, which can make it difficult to analyze. On the other hand, it is advantageous since its analysis provides information on the direct fetal exposure and its detection window is very wide, covering the second and third trimesters of pregnancy [11, 12].

Only three procedures have been published for the identification of several NPS in meconium [13–15]. So, to our knowledge, this is the first large multianalyte method for the identification of NPS in meconium. Besides, previous methods were developed with LC–MS/MS, which is in turn limited by the necessity to constantly update the analytical method with the new NPS emerging every day in the black market. In this sense, a QTOF technique by data-independent acquisition with sequential window acquisition of all theoretical fragment-ion mass spectra (SWATH) is more recommended, since it solves the limitations present in conventional mass spectrometers. This technique consists in a full scan of every detectable analyte present in the biological matrix by covering a wide mass range in several cycles. For each cycle, the instrument focuses on a small mass window of precursors and acquires MS/MS data from all precursors detected [16]. In addition, with this technique, new compounds can be added without changing the acquisition method, and retrospective analysis can be performed without the need to re-analyze the sample, since the SWATH acquisition collects MS and MS/MS information on each detectable peak. This is a great advantage as the sample quantity is sometimes scarce.

Thus, the aim of the present work was to develop and validate an analytical method for the determination of 137 NPS and metabolites in meconium by UHPLC-QTOF. Once validated, the method was applied to meconium specimens from cases in which fentanyl had been administered as epidural anesthesia at the time of delivery.

## Materials and methods

### Reagents and standards

All chemicals, including methanol, formic acid, dichloromethane, 2-propanol, ammonium hydroxide, and hydrochloric acid, were purchased from Sigma-Aldrich (Milan, Italy). Ultra-pure water was obtained using a Milli-Q® UF-Plus apparatus (Millipore, Bedford, MA, USA). All stock standard solutions were prepared in methanol at 1 mg/mL and stored at − 20 °C until used. Working solutions were prepared at the final concentration of 1000 ng/mL by dilution with methanol*.* SPE MCX cartridges (3 cm^3^, 60 mg) were acquired from Teknokroma (Barcelona, Spain).

Blank meconium specimens used for the preparation of the calibration curves were collected at the University Hospital of Vigo (Galicia, Spain) from newborns whose mothers were not suspicious of drug use during pregnancy. Meconium was collected at the hospital from newborn diapers up to 3 days after delivery, and stored in polypropylene containers at − 20 °C until analysis.

Moreover, 30 authentic meconium specimens were analyzed to prove the method applicability. These specimens were collected at the University Hospitals of Santiago de Compostela and Vigo (Galicia, Spain) from January 2012 to December 2015. Recruitment was done after delivery and mothers, who accepted to participate in the study and signed a written informed consent, were not paid for their participation. Real samples collection was approved by the Galician Clinical Research Ethics Committee (Xunta de Galicia, Spain; code number: 2011/203).

### Sample preparation

The sample was prepared following a previously published homogenization and extraction procedure [15]. Briefly, meconium (0.25 ± 0.02 g) was homogenized with 2 mL of methanol and 25 μL of the IStd solution at 1 μg/mL by sonication for 30 min. After centrifugation, the sample was evaporated to dryness under nitrogen at 45 °C. Then, the extract was reconstituted in 2 mL of 2% formic acid in H_2_O for solid-phase extraction (SPE). After cartridges conditioning (2 mL methanol + 2 mL water), the sample was loaded. Then, the column was subsequently washed with 2 mL of 2% formic acid in H_2_O and 2 mL of methanol/water/formic acid (47.5:47.5:5, v/v/v). After drying under vacuum for 10 min, analytes were eluted with 2 mL of dichloromethane/2-propanol/ammonium hydroxide (47.5:47.5:5, v/v/v). The final eluent of the SPE was evaporated to dryness with nitrogen at 45 °C and then reconstituted with 50 μL of methanol; finally, 5 μL were injected into the UHPLC-QTOF. Before each evaporation step, 50 μL of 1% HCl in methanol were added to prevent analyte evaporation.

### Instrumentation

UHPLC separation was performed on a Phenomenex Kinetex C18 column (100 × 2.1 mm, 1.7 μm) at 45 °C on the SCIEX ExionLC™ AC system. Mobile phases consisted of water (A) and acetonitrile (B), both with 5 mM of formic acid. The LC flow rate was 0.5 mL and the mobile phase eluted under the following linear gradient conditions: (A:B, v/v) isocratic elution at 95:5 for 0.5 min, from 95:5 to 5:95 in 7.5 min, isocratic elution at 5:95 for 0.5 min, and final re-equilibration for 2.5 min to the initial condition before each injection. Total run time was 10 min.

All analyses were performed using a quadrupole time-of-flight SCIEX X500R QTOF mass spectrometer (Sciex, Darmstadt, Germany) equipped with a Turbo VTM ion source operating in electrospray positive-ion mode. MS and MS/MS data were collected for each sample using SWATH™ Acquisition mode [17]. Data acquisition included a preliminary TOF-MS high-resolution scan followed by SWATH™ Acquisition using variable window setup (12 windows covering mass range from 150 to 465 *m*/*z* at 0.025 resolving power), resulting in a final cycle time of 0.564 s. Data were acquired using the SCIEX OS 1.5 Software.

### Method validation

Validation was performed according to a protocol published by Alladio et al. [18, 19]. Three calibration curves with 6 concentration levels were analyzed on 3 different days (3 × 3 × 6), and with these 54 data points, the main validation parameters were evaluated: calibration, intra- and inter-day precision and accuracy, limit of detection (LOD), selectivity, specificity, and carry-over. The method was only validated for semi-quantitative purposes.

#### Calibration

The heteroscedasticity of the data points was evaluated using an *F*-test integrated in the R routine. If the system was heteroscedastic, we selected the weighted model (linear or quadratic) that generates the smallest variance. Finally, the calibration model was calculated and the analysis of variance lack of fit (ANOVA-LoF) test was performed to verify it.

#### LOD

LODs were estimated using the Hubaux–Vox approach. When the analyte shows a quadratic trend, the highest levels of the calibration curve were eliminated until the trend was linear in order to calculate the LODs.

#### Precision and accuracy

Precision was evaluated using the coefficient of variation (%CV). Intra-day precision was evaluated calculating a calibration model for each day of validation, which was used to back-calculate the three experimental replicates performed the same day and then the %CV was calculated. While inter-day precision was computed by back calculating all the nine replicates using the comprehensive calibration curve. Intra- and inter-day precision were considered validated when the average of all the calibration levels was below 30%.

Intra-day accuracy was evaluated by a back-calculation of each data point using the calibration curves that did not include it. Two calibration curves were used to compute the calibration model using the R routine, then this model was used to back-calculate the third calibration curve and with the average of all the results, the overall bias was calculated, while inter-day accuracy was calculated in the same way but using the data point from the calibration curves of the other 2 days. Intra- and inter-day accuracy were considered validated when the average of the bias in all the calibration levels was below 20%.

Since the method was validated for semi-quantitative purposes, only inter-day precision was studied.

#### Matrix effect

Due to the potential variability in matrix composition obtained from different sources, matrix effect (ME) was investigated using 5 samples which were previously screened to confirm the absence of the analytes of interest [20]. ME studies were performed using the addition technique of the analytes and internal standards (IStd) to blank samples after their extraction. In this experiment, two sets of solutions of analytes and IStd were prepared at low (50 ng/g) and high (1000 ng/g) concentration levels within the method linear range: in methanol (set A) and in matrix extracts obtained from meconium samples (set B). The matrix effect (±%) can be explained as the ion suppression/enhancement and is calculated with the following formula:

$$ \mathrm{ME}\%=\left(\ \frac{\Big({\overline{\raisebox{1ex}{$ Ax$}\!\left/ \!\raisebox{-1ex}{$ Ais$}\right.\Big)}}_B}{{\overline{\left(\raisebox{1ex}{$ Ax$}\!\left/ \!\raisebox{-1ex}{$ Ais$}\right.\right)}}_A}-1\right)\times 100 $$

#### Selectivity and specificity

The presence of endogenous and exogenous interferences was checked by examining in the chromatogram the presence of interfering peaks with a signal/noise ratio above three around the retention time of the analytes. There must be an absence of interferences to validate the method.

#### Carry-over

The carry-over was studied by injecting 10 replicates of a blank sample meconium after the highest point of the calibration curve. It was considered validated when the signal was not greater than 20% of the LODs [21].

### Application to real specimens

Thirty paired meconium and maternal hair specimens from cases in which fentanyl had been administered as epidural anesthesia at the time of delivery (*n* = 27) or cases in which there was suspicion of drug abuse (*n* = 3) were studied.

Meconium specimens were analyzed with the present method to proof its applicability. Maternal hair specimens were analyzed using a previously published LC–MS/MS method that allows the determination of 35 analytes, including opioids, cocaine, amphetamines, cannabis, lysergic acid diethylamide, ketamine, scopolamine, antidepressants, benzodiazepines, and zolpidem [22]. Briefly, maternal hair (50 mg) was collected after delivery from the vertex posterior region, as close as possible to the scalp, and stored at room temperature. Maternal hair (8 cm) was divided into 3 segments corresponding with the 3 trimesters of pregnancy and individually analyzed: from 0 (root) to 2 cm, corresponding to the third trimester; from 3 to 5 cm, corresponding to the second trimester; and from 6 to 8 cm, corresponding to de first trimester.

## Results and discussion

The purpose of this publication was to develop and validate a screening method for the determination of NPS in meconium by UHPLC-QTOF. Other procedures for the detection of NPS in meconium have been published [13–15], but the number of target analytes was limited. Moreover, all these methods were developed in targeted LC-MS/MS, a valid analysis technique in most cases but with a limited capacity of detecting multianalyte when new compounds are constantly introduced into the market.

Meconium was prepared following a previously published homogenization and extraction procedure for the determination of 6 synthetic cathinones [15]. Therefore, it was demonstrated that this procedure could also be applied to the analysis of more than 100 very different compounds. Screening methods for the determination of NPS in other biological matrices have been published and, in all of them, the challenge of monitoring such many substances was observed [22–26]. As in the present method, all the mentioned analytical methods carried out an extraction procedure (liquid–liquid or solid-phase) [23–26] or even two separate extractions [22], paying special attention to the choice of the extraction solvent in order to achieve good recovery for all the compounds, which have varying chemical structures.

### Method validation

The total chromatographic run time was achieved in only 10 min and the retention time ranged between 0.7 min (psylocibin) and 8.03 min (AKB-48 APINACA). The method was validated for semi-quantitative purposes for all compounds listed in Tables 1 and 2, including 54 synthetic cannabinoids or metabolites; 49 synthetic cathinones, stimulants, hallucinogens, and metabolites; and 34 synthetic opioids and metabolites.

**Table 1:** Calibration model parameters, LOD, and LOQ for all the compounds

Compound	LOD (ng/g)	Calibration range (ng/g)	Weight	Model	Equation	Squared correlation coefficient (*r*^2^)
Synthetic cannabinoids						
5-Chloro-AB-PINACA	0.3	2–1000	1/*x*2	Linear	0.1108 *x* + 0.009756	0.9996
5-Chloro-TH-J018	0.25	10–1000	1/*x*2	Linear	0.03827 *x* − 0.0003938	0.9819
5-F-AB-PINACA	0.3	2–1000	1/*x*2	Linear	0.09489 *x* + 0.009723	0.9981
5-F-ADB	0.3	2–1000	1/*x*2	Linear	0.4424 *x* + 0.05326	0.9945
5-F-APINACA	1	10–750	1/*x*	Quadratic	− 0.2416 *x*^2^ + 4.825 *x* + 0.3457	0.7398
5-F-APP PICA	0.4	2–750	1/*x*2	Linear	0.4928 *x* + 0.08252	0.9976
5-F-APP PINACA	0.3	10–1000	1/*x*2	Linear	0.1738 *x* − 0.00544	0.9926
5-F-CUMYL PINACA	0.4	2–750	1/*x*2	Linear	0.6808 *x* + 0.1156	0.9826
5-F NNEI 2′-naphthyl isomer	0.7	2–750	1/*x*	Linear	0.3296 *x* + 0.06084	0.9957
AB-CHMINACA	0.3	2–1000	1/*x*2	Linear	0.2223 *x* + 0.02269	0.9965
AB-FUBINACA	0.5	2–750	1/*x*	Linear	0.1126 *x* + 0.01023	0.9982
AB-PINACA	0.25	10–1000	1/*x*2	Linear	0.2159 *x* + 0.005061	0.9989
ADB-FUBINACA	0.4	2–750	1/*x*	Linear	0.1153 *x* + 0.008009	0.9971
ADBICA	0.6	2–750	1/*x*	Linear	0.336 *x* + 0.04333	0.997
ADB-PINACA	0.3	2–1000	1/*x*2	Linear	0.2111 *x* + 0.02225	0.9987
AKB-48 APINACA	0.3	2–750	1/*x*	Quadratic	− 0.003394 *x*^2^ + 0.1206 *x* + 0.01288	0.9915
AM-1220	0.1	10–1000	1/*x*2	Quadratic	0.005469 *x*^2^ + 0.296 *x* − 0.01568	0.9964
AM-2201	0.6	2–750	1/*x*	Linear	0.1377 *x* + 0.01795	0.9991
AM-2233	0.2	10–1000	1/*x*2	Linear	1.258 *x* − 0.06359	0.993
AM-694	0.3	2–1000	1/*x*2	Linear	0.07424 *x* + 0.007622	0.9981
APP-FUBINACA	0.3	2–750	1/*x*2	Linear	0.1132 *x* + 0.009388	0.991
CUMYL-PeGACLONE	0.3	2–750	1/*x*2	Linear	1.543 *x* + 0.1826	0.8529
JWH-007	0.5	2–750	1/*x*	Linear	0.4384 *x* + 0.05555	0.9986
JWH-015	0.4	2–750	1/*x*	Linear	0.4562 *x* + 0.04051	0.9957
JWH-016	0.6	2–1000	1/*x*	Linear	0.6233 *x* + 0.07023	0.9987
JWH-018	0.6	2–1000	1/*x*	Linear	0.6247 *x* + 0.07083	0.9987
JWH-019	0.3	2–750	1/*x*	Quadratic	− 0.002751 *x*^2^ + 0.1765 *x* + 0.01267	0.9959
JWH-020	0.3	10–1000	1/*x*	Quadratic	− 0.000976 *x*^2^ + 0.0729 *x* − 0.001472	0.9934
JWH-073	0.1	2–750	1/*x*2	Quadratic	− 0.02009 *x*^2^ + 0.6237 *x* + 0.001378	0.899
JWH-081	0.5	2–750	1/*x*	Linear	0.3621 *x* + 0.09221	0.9973
JWH-098	0.1	2–500	1/*x*2	Linear	0.2778 *x* + 0.01005	0.9837
JWH-122	0.3	2–750	1/*x*	Quadratic	− 0.002569 *x*^2^ + 0.1695 *x* + 0.01332	0.9979
JWH-147	0.04	2–500	1/*x*2	Quadratic	− 0.008044 *x*^2^ + 0.2343 *x* − 0.0004362	0.9853
JWH-203	0.3	2–750	1/*x*2	Linear	0.1766 *x* + 0.01617	0.9994
JWH-210	0.3	2–1000	1/*x*2	Linear	0.109 *x* + 0.01019	0.9964
JWH-250	0.3	2–500	1/*x*2	Linear	16.36 *x* + 1.573	0.8404
JWH-251	0.3	2–750	1/*x*2	Linear	0.4398 *x* + 0.05204	0.998
JWH-302	0.3	2–750	1/*x*2	Linear	0.2207 *x* + 0.02539	0.9985
JWH-307	0.1	2–500	1/*x*2	Linear	0.2791 *x* + 0.01007	0.9839
JWH-398	0.2	10–1000	1/*x*2	Quadratic	− 0.0008424 *x*^2^ + 0.05617 *x* − 0.0001841	0.9951
MAB-CHMINACA	0.3	2–750	1/*x*2	Linear	0.2734 *x* + 0.02795	0.997
MAM-2201	0.3	2–500	1/*x*2	Linear	0.4105 *x* + 0.04215	0.9957
MDMB-CHMICA	1.1	50–1000	1/*x*2	Quadratic	− 0.0002132 *x*^2^ + 0.00765 *x* + 0.0005146	0.973
MDMB-CHMINACA	0.5	2–500	1/*x*	Quadratic	− 0.1915 *x*^2^ + 3.181 *x* + 0.3453	0.8686
MMB-2201	0.4	2–750	1/*x*2	Linear	0.8861 *x* + 0.152	0.9845
PB-22	0.4	2–750	1/*x*	Quadratic	− 0.03439 *x*^2^ + 1.358 *x* + 0.1977	0.9927
RCS-4	0.5	2–750	1/*x*	Linear	0.6175 *x* + 0.0641	0.998
RCS-8	0.4	2–750	1/*x*	Linear	0.334 *x* + 0.0317	0.9999
STS-135	0.3	2–750	1/*x*2	Linear	0.3803 *x* + 0.0396	0.9314
UR-144	0.3	2–750	1/*x*	Linear	0.117 *x* + 0.006706	0.9999
UR-144-5-OH	0.3	2–750	1/*x*2	Linear	0.415 *x* + 0.05968	0.9903
WIN-48	0.2	10–750	1/*x*2	Linear	0.4263 *x* + 0.04025	0.9742
WIN-55	0.2	10–750	1/*x*2	Linear	0.4956 *x* − 0.02026	0.9757
XLR-11	0.3	2–750	1/*x*2	Linear	0.2441 *x* + 0.02587	0.9988
Synthetic cathinones and hallucinogens						
25B-NBOMe	1	50–1000	1/*x*2	Quadratic	− 0.5271 *x*^2^ + 31.84 *x* + 25.43	0.94
25C-NBOMe	0.7	2–750	1/*x*	Quadratic	− 0.802 *x*^2^ + 26.85 *x* + 5.498	0.9106
25H-NBOMe	0.3	2–750	1/*x*	Quadratic	− 1.207 *x*^2^ + 42.43 *x* + 13.83	0.9404
25I-NBOMe	2.3	2–1000	1	Quadratic	− 0.9354 *x*^2^ + 43.96 *x* + 9.964	0.9641
2C-B	0.7	2–1000	1/*x*	Quadratic	− 0.003879 *x*^2^ + 0.1796 *x* + 0.03881	0.9913
2C-P	0.4	10–1000	1/*x*2	Quadratic	− 0.009927 *x*^2^ + 0.3245 *x* + 0.09366	0.8786
3-4-DMMC	0.6	10–1000	1/*x*2	Linear	0.1401 *x* + 0.006774	0.9837
4-Acetoxy-DiPT	0.1	10–1000	1/*x*2	Linear	0.1397 *x* + 0.002963	0.9888
4-Acetoxy-DMT	0.2	10–1000	1/*x*2	Quadratic	− 0.01964 *x*^2^ + 0.7116 *x* + 0.0764	0.9637
4-FA	0.8	2–750	1/*x*	Linear	0.5245 *x* + 0.1465	0.9968
4-F-Methcathinone	1.2	50–1000	1/*x*2	Linear	0.2239 *x* + −0.02987	0.9761
4-MEC	0.4	10–1000	1/*x*2	Quadratic	− 0.006333 × 2 + 0.1926 *x* + 0.02581	0.9624
5-EAPB	0.1	10–1000	1/*x*2	Quadratic	− 0.1092 *x*^2^ + 4.679 *x* + 0.1457	0.9896
5-MAPB	0.3	10–1000	1/*x*2	Linear	0.1197 *x* + 0.00334	0.987
5-Methoxy AMT	0.7	10–1000	1/*x*2	Linear	0.09593 *x* + 0.01237	0.9896
5-Methoxy DALT	0.9	2–1000	1/*x*	Quadratic	0.01582 *x*^2^ + 0.7615 *x* + 0.1362	0.9778
5-Methoxy DMT	0.2	2–750	1/*x*2	Linear	0.6774 *x* + 0.1027	0.9972
5-Methoxy DiPT	0.6	2–750	1/*x*	Linear	0.7815 *x* + 0.05913	0.996
5-OH-tryptophan	1.2	2–750	1/*x*	Quadratic	− 0.01051 *x*^2^ + 0.2862 *x* + 0.05953	0.9695
6-APB	0.8	2–750	1/*x*	Linear	0.4281 *x* + 0.1081	0.9941
Buphedrone	2	50–1000	1/*x*2	Linear	1.026 *x* − 0.3712	0.977
Butylone	0.4	10–1000	1/*x*2	Quadratic	− 0.006513 *x*^2^ + 0.2355 *x* + 0.02021	0.9905
DMT	0.3	2–750	1/*x*2	Linear	6.705 *x* + 0.6186	0.9945
Ethylone	0.5	10–1000	1/*x*	Quadratic	− 0.01085 *x*^2^ + 0.6566 *x* + 0.1148	0.9955
Ethylphenidate	1.1	2–1000	1/*x*2	Quadratic	− 0.04227 *x*^2^ + 2.061 *x* + 0.4308	0.9704
Ethyltryptamine	0.2	2–1000	1/*x*	Quadratic	− 0.004067 *x*^2^ + 0.5112 *x* + 0.01163	0.994
Harmine	1.3	2–1000	1/*x*	Quadratic	− 0.007595 *x*^2^ + 0.3158 *x* + 0.08406	0.9676
Ketamine	0.7	2–1000	1/*x*	Linear	0.5291 *x* + 0.1123	0.9987
LSD	0.5	2–1000	1/*x*	Linear	0.464 *x* + 0.05979	0.9962
mCPP	0.7	2–1000	1/*x*	Linear	0.8268 *x* + 0.1603	0.9993
MDPV	1.6	10–1000	1/*x*	Linear	0.385 *x* + 0.01433	0.9946
Mephedrone	0.6	50–1000	1/*x*2	Linear	0.3382 *x* + −0.05036	0.9907
Mescaline	0.3	10–1000	1/*x*2	Quadratic	− 0.007273 *x*^2^ + 0.2077 *x* + 0.04462	0.9546
Methedrone	0.3	10–1000	1/*x*2	Linear	0.2777 *x* + 0.0299	0.9877
Methylone	0.4	10–1000	1/*x*2	Quadratic	− 0.01108 *x*^2^ + 0.4579 *x* + 0.03933	0.98
Mexedrone	2.3	50–1000	1/*x*2	Linear	0.03122 *x* + 0.04465	0.9656
Mitragynine	0.1	10–1000	1/*x*2	Quadratic	− 0.002645 *x*^2^ + 0.2982 *x* + 0.004006	0.9931
N-Ethylcathinone	2.4	50–1000	1/*x*2	Linear	0.01482 *x* − 0.004482	0.9662
N-Ethylpentylone	0.2	10–1000	1/*x*2	Quadratic	− 0.008614 *x*^2^ + 0.3912 *x* + 0.02911	0.9862
PCP	1.6	2–750	1	Linear	0.6047 *x* + 0.1403	0.9977
4-MeO-PCP	0.7	10–1000	1/*x*	Linear	0.5299 *x* + 0.09312	0.9983
Pentedrone	0.4	10–1000	1/*x*2	Linear	0.1635 *x* + 0.004438	0.9824
Pentylone	0.2	10–1000	1/*x*	Quadratic	− 0.004814 *x*^2^ + 0.3041 *x* + 0.0185	0.9898
PMA	0.8	2–750	1/*x*	Linear	0.3182 *x* + 0.08759	0.9979
PMMA	0.9	2–750	1/*x*	Linear	0.6716 *x* + 0.201	0.9942
Psilocin	0.4	10–1000	1/*x*2	Linear	0.1752 *x* + 0.00455	0.9967
Ritanilic acid	0.3	10–1000	1/*x*2	Linear	0.2817 *x* + 0.05225	0.9991
Trazodone	0.9	2–1000	1/*x*	Linear	0.5529 *x* + 0.1541	0.9939
α-PVP	0.2	10–1000	1/*x*2	Linear	0.2971 *x* + 0.01806	0.9929
Fentanyl analogs and synthetic opioids						
3-Methylnorfentanyl	0.1	10–1000	1/*x*2	Quadratic	− 0.001644 *x*^2^ + 0.1505 *x* − 0.001454	0.9933
4-ANPP	0.7	2–1000	1/*x*	Quadratic	− 0.002447 *x*^2^ + 0.1769 *x* + 0.03207	0.9942
4-F-Butyrylfentanyl	0.1	10–750	1/*x*2	Linear	0.3749 *x* + 0.007014	0.9971
4-Methyl fentanyl	0.1	10–750	1/*x*2	Quadratic	− 0.003541 *x*^2^ + 0.283 *x* + 0.00173	0.9987
Acetyl fentanyl	0.1	10–1000	1/*x*2	Quadratic	− 0.02346 *x*^2^ + 0.7346 *x* + 0.02761	0.9866
Acetyl norfentanyl	0.2	10–750	1/*x*2	Quadratic	− 0.00545 *x*^2^ + 0.2208 *x* + 0.009908	0.9802
Acrylfentanyl	0.2	10–750	1/*x*2	Quadratic	− 0.02201 *x*^2^ + 0.6496 *x* + 0.01222	0.951
AH-7921	0.2	10–1000	1/*x*2	Linear	0.1083 *x* − 0.0008527	0.9945
Alfentanyl	0.3	10–750	1/*x*2	Quadratic	− 0.02268 *x*^2^ + 0.4726 *x* + 0.05711	0.838
Butyrylfentanyl	0.1	10–750	1/*x*2	Quadratic	− 0.01204 *x*^2^ + 0.5849 *x* − 0.004432	0.995
Butyryl fentanyl carboxy metabolite	0.7	2–750	1/*x*	Linear	0.3062 *x* + 0.06538	0.9955
Butyryl norfentanyl	0.5	2–750	1/*x*	Quadratic	− 0.006895 *x*^2^ + 0.3085 *x* + 0.04378	0.988
Carfentanyl	0.1	10–1000	1/*x*2	Quadratic	− 0.002613 *x*^2^ + 0.1908 *x* + 0.00934	0.995
Cyclopropylfentanyl	0.1	10–750	1/*x*2	Quadratic	− 0.005917 *x*^2^ + 0.4781 *x* + 0.003248	0.9983
Despropionyl p-fluorofentanyl	0.3	10–1000	1/*x*2	Linear	0.1468 *x* + 0.0009262	0.9919
Fentanyl	2.2	10–1000	1	Quadratic	− 0.006104 *x*^2^ + 0.4574 *x* + 0.08624	0.9923
Furanylfentanyl	0.2	10–750	1/*x*2	Quadratic	− 0.01427 *x*^2^ + 0.6959 *x* + 0.01409	0.9918
Furanylnorfentanyl	0.2	10–750	1/*x*2	Quadratic	− 0.01182 *x*^2^ + 0.3486 *x* + 0.007127	0.9784
Hydrocodone	0.5	10–1000	1/*x*2	Linear	0.02651 *x* + 0.001894	0.9932
Methoxyacetyl norfentanyl	0.1	10–750	1/*x*2	Quadratic	− 0.004828 *x*^2^ + 0.1743 *x* + 0.01007	0.9957
MT-45	0.3	10–750	1/*x*2	Linear	0.2524 *x* + 0.03461	0.9803
Norfentanyl	0.5	50–1000	1/*x*2	Quadratic	− 0.009264 *x*^2^ + 0.4635 *x* + 0.1825	0.9847
Ocfentanyl	0.1	10–1000	1/*x*2	Quadratic	− 0.01969 *x*^2^ + 0.776 *x* + 0.0339	0.9816
OH-fentanyl	0.1	10–1000	1/*x*2	Quadratic	− 0.00299 *x*^2^ + 0.17 *x* + −8.721*e*−06	0.9912
Thiofentanyl	0.2	10–1000	1/*x*2	Quadratic	− 0.003418 *x*^2^ + 0.1964 *x* + 0.0002254	0.994
Oxycodone	0.6	10–1000	1/*x*2	Linear	0.1192 *x* + 0.01877	0.9919
Phenylacetyl fentanyl	0.5	2–1000	1/*x*	Linear	0.3061 *x* + 0.03762	0.9996
4-Phenylfentanyl	0.1	10–750	1/*x*2	Linear	0.5208 *x* + 0.01129	0.9975
Remifentanyl	0.6	2–750	1/*x*2	Quadratic	− 0.009414 *x*^2^ + 0.2978 *x* + 0.0546	0.9577
Sufentanyl	0.1	10–1000	1/*x*2	Quadratic	− 0.001314 *x*^2^ + 0.08714 *x* − 0.001218	0.9985
Tramadol	0.2	10–750	1/*x*2	Quadratic	− 0.03904 *x*^2^ + 1.088 *x* + 0.0874	0.9322
U47700	0.3	10–750	1/*x*2	Quadratic	− 0.01362 *x*^2^ + 0.3489 *x* + 0.02041	0.9039
Valeryl fentanyl carboxy metabolite	0.1	10–1000	1/*x*2	Quadratic	− 0.002608 *x*^2^ + 0.1907 *x* + 0.009362	0.995
β-Phenylfentanyl	0.1	10–750	1/*x*2	Quadratic	− 0.008265 *x*^2^ + 0.3967 *x* − 0.002192	0.974

**Table 2:** Inter-day precision, expressed in terms of CV. Acceptable results are expected in the range ± 30%. Values exceeding 30% are reported in bold

Compound	Calibration level (CV %)					
	Inter-d					
	1	2	3	4	5	6
Synthetic cannabinoids						
5-Chloro-AB-PINACA	15.7	28.5	0.5	14.4	2.1	0.2
5-Chloro-TH-J018	0.1	3.5	9.4	11.4	6.5	12
5-F-AB-PINACA	15.5	**30.1**	0.9	18.2	6.1	1.5
5-F-ADB	4.9	29.4	11.5	23.6	1.1	11.8
5-F-APINACA	0.5	0.1	0	10.2	**40.8**	**57.7**
5-F-APP PICA	10.6	**30.5**	14	**33.5**	3.7	2.5
5-F-APP PINACA	0.9	11.3	8.4	15.8	2.5	11.4
5-F-CUMYL PINACA	0.9	2.6	22.9	25.9	15.9	29.5
5-F NNEI 2′-naphthyl isomer	7.4	**37**	21	27	2.6	6.2
AB-CHMINACA	5.9	**33**	2.2	20.4	4.1	8.4
AB-FUBINACA	28.7	**48.5**	4.1	19.4	4.4	0.7
AB-PINACA	1.2	2.2	18.3	6.9	2.5	5.6
ADB-FUBINACA	**32.4**	**38.8**	8.4	8.5	10.5	3.6
ADBICA	28.4	22.3	23.5	**34.6**	5.2	2.3
ADB-PINACA	3.4	18.6	2.1	18.8	3.9	2.4
AKB-48 APINACA	15	**34**	15	11	15	–
AM-1220	0.1	0	0.4	2.6	6.6	4
AM-2201	25.6	**36.2**	6.4	19	1.9	0.4
AM-2233	0.8	2.9	3.5	0.2	0.6	4.6
AM-694	4.9	26.2	16.7	**39.1**	0.9	2.1
APP-FUBINACA	9.1	**45.9**	7	10.9	**30.2**	2.7
CUMYL-PeGACLONE	0.9	8	3.3	24.3	23.6	**44**
JWH-007	**44.6**	**53.4**	1.2	9.8	4	1.8
JWH-015	**35.5**	**42.1**	11.4	9.4	13	4.3
JWH-016	10.5	**32.9**	15.8	**32.1**	1.6	2.8
JWH-018	11.3	**32.7**	16	**32.4**	1.6	2.9
JWH-019	22.9	**30.2**	3	8	3.7	0.4
JWH-020	0.2	2.5	8.8	15.9	7	5
JWH-073	0.1	2.3	10.7	1.2	19.1	23
JWH-081	15	**48.4**	10.4	28.6	5.2	0.4
JWH-098	3.4	15.3	10.7	3.5	3.1	22.9
JWH-122	16.3	29.8	6.2	11.6	8.2	4.4
JWH-147	1.5	9	3.9	9.2	6.9	1.9
JWH-203	4.4	24	5.6	16	1.9	0.1
JWH-210	8.2	**45**	8.7	**30.6**	6.4	4.1
JWH-250	0.1	1.7	17.8	26.2	–	–
JWH-251	2.9	19.6	16.7	19.6	8.4	11.2
JWH-302	3.9	20.9	1.3	21.2	3.4	0.6
JWH-307	3.5	15.5	10.7	3.5	3.3	22.9
JWH-398	0.3	4.4	8.8	11	3.6	5
MAB-CHMINACA	5.4	**31.7**	8.1	**32.4**	6.3	8
MAM-2201	7.7	**37.1**	15.7	7.3	10.5	27.3
MDMB-CHMICA	4.8	10.7	7.2	29	9.9	–
MDMB-CHMINACA	**32.9**	**39.4**	2.7	6.4	3.5	**33.1**
MMB-2201	0.8	1.6	19.3	18.5	10.1	25.4
PB-22	15.2	26.3	2.5	13.1	10.4	11
RCS-4	16.9	23.6	4.3	14	4.8	1.9
RCS-8	**32**	**37.4**	0.4	5.7	0.6	0.2
STS-135	6.6	**35.2**	1.5	27.4	23.5	20.5
UR-144	19.5	24.7	0.6	6	0.8	0.7
UR-144-5-OH	0.7	10.7	26.7	23.8	15.5	25
WIN-48	0.1	0.4	4.3	2	14.8	13
WIN-55	0.9	1	14	7.2	11.6	16.5
XLR-11	5.5	**31.3**	7	2.5	2.3	2.3
Synthetic cathinones and hallucinogens						
25B-NBOMe	2.1	8.1	14.5	8	14.6	14
25C-NBOMe	8.2	19.1	11.6	1.6	4.5	**30.2**
25H-NBOMe	22.8	22.4	6.4	12.7	11.2	15.6
25I-NBOMe	20.9	12.5	0.1	9.6	15.8	7.5
2C-B	18.8	28.7	8.3	2.9	5.2	14.6
2C-P	2.6	17.9	1.6	25.4	10	–
3-4-DMMC	1.1	16.1	21.1	4.4	2.7	13.4
4-Acetoxy-DiPT	0.2	1.1	1.3	3.3	7.1	8.4
4-Acetoxy-DMT	1	7.5	4.8	5.1	13.9	–
4-FA	10.8	23.6	23.1	12.9	3.6	5.3
4-F-Methcathinone	2.1	10.1	17.7	5.1	17.4	2.8
4-MEC	0.5	0.1	8	23.2	22.8	–
5-EAPB	1.6	8.7	2.5	29.6	10.1	–
5-MAPB	0.6	4.5	3.4	7.6	2.9	10.8
5-Methoxy AMT	0.5	13.1	23.4	16.1	6	0.7
5-Methoxy DALT	8.2	11.5	2.1	1.5	1.5	3.6
5-Methoxy DMT	3.5	13.7	17.9	11.9	21	18.9
5-Methoxy DiPT	2.4	25.1	16.4	6.3	5.3	5.1
5-OH-tryptophan	20.2	9.3	14.2	0.3	13.1	–
6-APB	1.3	**41.8**	25.9	19.2	4.8	6.9
Buphedrone	5.3	14.9	15	9.6	6.3	10.6
Butylone	1.1	5.7	2.7	18.8	21.8	–
DMT	5.6	**33.4**	12.9	28.7	2.5	11.3
Ethylone	22.2	11.7	17.2	7.6	4.7	7.8
Ethylphenidate	5.3	4	3.5	7.3	7.5	4.6
Ethyltryptamine	4.8	11	5	13.5	6	3.5
Harmine	9.2	8.7	2.9	1.3	7.5	–
Ketamine	**50.3**	**48.8**	4.3	0.3	5.1	1.9
LSD	0.1	13.5	6.1	7.1	5.2	4.8
mCPP	28.6	55.9	9.9	20.6	2.7	0.7
MDPV	0	0.7	1.5	0.3	5	6
Mephedrone	1.5	4.8	4	6.4	9.6	2.5
Mescaline	1.8	12.7	5	21.9	**38.2**	–
Methedrone	2.5	9.9	6.8	0.2	0.3	14.3
Methylone	0.1	1.6	5.8	11.6	0.1	–
Mexedrone	2.2	12.8	**30.8**	13.3	13.9	7.1
Mitragynine	0.8	7.8	8.3	0.3	4.6	3.1
N-Ethylcathinone	11.2	15.6	**32.3**	17.8	20.3	1.5
N-Ethylpentylone	0.6	3.3	0.1	7.6	2.6	–
PCP	34.6	49.8	3.8	14.4	5.1	2
4-MeO-PCP	27.1	15	15.3	4	2.9	2.2
Pentedrone	0	4.2	8.8	1.6	8.3	11.3
Pentylone	0.2	1.3	0.5	5.6	4.3	0.4
PMA	4.4	**31.7**	19.5	9	3.1	4.2
PMMA	12.1	**33.6**	29.9	17.8	4.7	7
Psilocin	0.4	2.6	1.9	7.7	5.7	2.1
Ritanilic acid	0.5	0.8	7.4	1.8	4.8	3
Trazodone	**34.2**	14.8	19.5	3	3.9	7.1
α-PVP	0.6	4.8	3.9	2.1	5	7.3
Fentanyl analogs and synthetic opioids						
3-Methylnorfentanyl	0.1	1.7	4.5	8.4	6.7	0.6
4-ANPP	14.1	4.6	9.9	10.3	0.4	2.2
4-F-Butyrylfentanyl	0.5	1	3.8	1.9	7.1	0.9
4-Methyl fentanyl	0.1	2.6	5.7	2	4.7	3.9
Acetyl fentanyl	0	2.1	6.4	2.6	13.6	–
Acetyl norfentanyl	0.6	5	2.7	6	7.1	4.1
Acrylfentanyl	0.1	0.7	1.3	7.7	15.2	–
AH-7921	0.7	2.4	3.9	3.4	4.6	6.5
Alfentanyl	0.5	3.4	0.5	2.6	**38.5**	**45.1**
Butyrylfentanyl	0.7	5.2	2.8	0.6	7.5	9
Butyryl fentanyl carboxy metabolite	**47.1**	15.9	**30.7**	11.1	6.1	4.7
Butyryl norfentanyl	5.7	0.9	8.3	4.1	11.6	8.4
Carfentanyl	0.1	2.2	5.1	7.7	3.3	2.2
Cyclopropylfentanyl	0.3	4.1	6.9	1.1	5.9	4.3
Despropionyl p-fluorofentanyl	0.8	8.5	28.2	5.3	0.8	12.7
Fentanyl	**71.5**	18.2	2.5	1.4	1.8	0.6
Furanylfentanyl	0.1	0	0.6	3.9	11.8	10.6
Furanylnorfentanyl	0.5	1.3	5.5	4.9	19.9	–
Hydrocodone	4	29.4	17.2	3.2	11.4	0.1
Methoxyacetyl norfentanyl	1.5	6.2	11.4	21.8	10.9	–
MT-45	1.6	0.5	11.9	20.1	15.6	15.2
Norfentanyl	1.6	3.2	4.7	13.5	2.6	18.2
Ocfentanyl	0.4	0.8	4.6	57.7	11.4	–
OH-fentanyl	0.1	2.8	6.6	10.3	4.2	5.5
Thiofentanyl	0.5	1.2	3.6	12.1	3.5	9.1
Oxycodone	5.8	**33.8**	4.5	15.9	8.5	1.1
Phenylacetyl fentanyl	**45.3**	**51.2**	0.3	4.9	2.5	1.3
4-Phenylfentanyl	1.1	2.8	6.3	4.3	8.6	3.7
Remifentanyl	25.5	**55.9**	3.1	8.1	2.2	0.5
Sufentanyl	0.7	4.1	0	6.8	5.1	12
Tramadol	0.3	1.8	0.8	8.4	14.2	–
U47700	0.4	2.2	2.1	14.9	24.9	–
Valeryl fentanyl carboxy metabolite	0	2.2	5.1	7.7	3.3	2.1
β-Phenylfentanyl	0.9	1.5	1.9	5.5	24.9	**31.9**

For all analytes, the calibration data points proved to have heteroscedastic distribution, using 1, 1/*x* and 1/*x*2 as a weighting factor depending on the analyte (Table 1). The calibration curves were studied in the ranges between 2, 10, or 50 ng/g and 750 or 1000 ng/g, depending on the analyte. The calibration curves for the following analytes proved quadratic within the calibration range: 5-F-APINACA, AKB-48 APINACA, AM-1220, JWH-019, JWH-020, JWH-073, JWH-122, JWH-147, JWH-398, MDMB-CHMICA, MDMB-CHMINACA,PB-22, 25B-NBOMe, 25C-NBOMe, 25H-NBOMe, 25I-NBOMe, 2-CB, 2-CP, 4-Acetoxy-DMT, 4-MEC, 5-EAPB, 5-methoxy DALT, 5-OH-tryptophan, butylone, ethylone, ethylphenidate, ethyltryptamine, harmine, mescaline, methylone, mitragynine, N-ethylpentylone, pentylone, 3-methylnorfentanyl, 4-ANPP, 4-methyl fentanyl, acetyl fentanyl, acetyl norfentanyl, acrylfentanyl, alfentanyl, butyrylfentanyl, butyryl norfentanyl, carfentanyl, cyclopropylfentanyl, fentanyl, furanylfentanyl, furanylnorfentanyl, methoxyacetyl norfentanyl, norfentanyl, ocfentanyl, OH-fentanyl, thiofentanyl, remifentanyl, sufentanyl, tramadol, U47700, valeryl fentanyl carboxy metabolite, and β-phenylfentanyl, while for the rest of the analytes a linear fitting was more suitable. All the equations for the final calibration models are also reported in Table 1.

LOD ranged between 0.04 and 2.4 ng/g (Table 1), while LOD described by other authors [12–14] were much higher (0.5–10 ng/g). Moreover, Pichini et al. [13] method uses 0.5 g of meconium, which is twice what was used in this method, while Nemeškalová et al. [14] and López-Rabuñal et al. [15] methods use a similar amount of meconium. Using a lower amount of sample is crucial because it allows additional drug analysis when the specimen quantity is limited.

Inter-day precision (expressed as percent variation coefficient, CV %) were found to be between 0.0 and 71.5 (Table 2). Matrix effect ranged from − 70 to 72% for synthetic cannabinoids, − 89 to 71% for synthetic cathinones and hallucinogens, and − 88 to 110% for fentanyl analogous and synthetic opioids. Matrix effect varied from signal suppression to high signal enhancement due to coeluting endogenous substances, and significant values were obtained for most compounds (only matrix effect between − 20 and 20% are considered negligible). Matrix effect results are shown in Table 3.

**Table 3:** Matrix effect at low (50 ng/g) and high (1000 ng/g) concentration levels

Compound	Matrix effect (*n* = 5)	
	(±%)	
	50 ng/g	1000 ng/g
Synthetic cannabinoids		
5-Chloro-AB-PINACA	− 45	− 46
5-Chloro-TH-J018	− 21	16
5-F-AB-PINACA	− 38	− 40
5-F-ADB	− 23	− 22
5-F-APINACA	− 43	− 47
5-F-APP PICA	− 32	− 39
5-F-APP PINACA	− 40	− 51
5-F-CUMYL PINACA	− 70	− 33
5-F NNEI 2′-naphthyl isomer	− 70	− 35
AB-CHMINACA	− 36	− 27
AB-FUBINACA	− 49	2
AB-PINACA	− 32	− 43
ADB-FUBINACA	− 37	− 40
ADBICA	− 26	− 30
ADB-PINACA	− 17	− 28
AKB-48 APINACA	− 49	− 25
AM-1220	− 16	− 22
AM-2201	− 46	− 45
AM-2233	− 48	− 40
AM-694	− 26	− 24
APP-FUBINACA	− 60	− 54
CUMYL-PeGACLONE	33	72
JWH-007	− 33	− 40
JWH-015	− 31	− 30
JWH-016	24	29
JWH-018	− 18	− 12
JWH-019	− 39	− 13
JWH-020	− 45	− 18
JWH-073	− 14	− 17
JWH-081	− 6	− 5
JWH-098	− 26	8
JWH-122	− 39	− 13
JWH-147	− 42	− 36
JWH-203	− 38	− 22
JWH-210	− 49	− 46
JWH-250	− 27	− 23
JWH-251	− 6	− 12
JWH-302	− 39	− 18
JWH-307	− 26	− 30
JWH-398	− 50	− 40
MAB-CHMINACA	− 19	− 15
MAM-2201	− 10	4
MDMB-CHMICA	− 26	− 23
MDMB-CHMINACA	− 43	− 33
MMB-2201	17	20
PB-22	− 16	− 18
RCS-4	− 20	13
RCS-8	− 27	7
STS-135	− 60	− 23
UR-144	− 44	− 8
UR-144-5-OH	− 28	17
WIN-48	− 50	− 50
WIN-55	− 43	− 45
XLR-11	− 37	− 36
Synthetic cathinones and hallucinogens		
25B-NBOMe	− 81	− 50
25C-NBOMe	− 80	− 55
25H-NBOMe	− 75	− 41
25I-NBOMe	− 80	− 52
2C-B	28	50
2C-P	48	46
3-4-DMMC	− 78	− 72
4-Acetoxy-DiPT	− 66	− 89
4-Acetoxy-DMT	− 74	− 70
4-FA	− 20	− 21
4-F-Methcathinone	1	− 3
4-MEC	− 52	− 50
5-EAPB	− 77	− 60
5-MAPB	− 76	− 63
5-Methoxy AMT	− 78	− 80
5-Methoxy DALT	− 77	− 73
5-Methoxy DMT	− 78	− 71
5-Methoxy DiPT	− 78	− 71
5-OH-Tryptophan	− 61	− 51
6-APB	− 75	− 74
Buphedrone	− 13	− 5
Butylone	− 31	− 13
DMT	13	45
Ethylone	31	71
Ethylphenidate	− 68	− 50
Ethyltryptamine	− 72	− 70
Harmine	− 71	− 71
Ketamine	6	8
LSD	− 75	− 62
mCPP	− 77	− 71
MDPV	− 65	− 52
Mephedrone	− 67	− 65
Mescaline	46	70
Methedrone	− 39	− 34
Methylone	− 40	− 42
Mexedrone	− 70	− 65
Mitragynine	4	25
N-Ethylcathinone	19	8
N-Ethylpentylone	20	24
PCP	− 4	3
4-MeO-PCP	− 14	− 4
Pentedrone	− 3	6
Pentylone	28	30
PMA	− 16	1
PMMA	27	8
Psilocin	32	32
Ritanilic acid	9	34
Trazodone	9	30
α-PVP	2	9
Fentanyl analogous and synthetic opioids		
3-Methylnorfentanyl	− 11	− 12
4-ANPP	− 76	− 71
4-F-Butyrylfentanyl	− 78	− 68
4-Methyl fentanyl	− 78	− 75
Acetyl fentanyl	− 70	− 57
Acetyl norfentanyl	− 4	− 9
Acrylfentanyl	− 12	1
AH-7921	3	9
Alfentanyl	68	110
Butyrylfentanyl	4	18
Butyryl fentanyl carboxy metabolite	19	2
Butyryl norfentanyl	− 72	− 54
Carfentanyl	1	− 3
Cyclopropylfentanyl	− 1	10
Despropionyl p-fluorofentanyl	28	33
Fentanyl	27	38
Furanylfentanyl	78	110
Furanylnorfentanyl	− 1	11
Hydrocodone	16	− 14
Methoxyacetyl norfentanyl	25	48
MT-45	− 84	− 74
Norfentanyl	− 50	− 16
Ocfentanyl	8	34
OH-Fentanyl	− 77	− 78
Thiofentanyl	− 76	− 76
Oxycodone	− 11	− 5
Phenylacetyl fentanyl	− 73	− 72
4-Phenylfentanyl	− 76	− 66
Remifentanyl	11	74
Sufentanyl	− 47	− 9
Tramadol	− 60	− 57
U47700	− 74	− 63
Valeryl fentanyl carboxy metabolite	− 78	− 70
β-Phenylfentanyl	− 77	− 88

No endogenous and/or exogenous interferences with a signal/noise ratio above 3 were detected around the retention time of the analytes; therefore, selectivity and specificity were verified for all analytes. Finally, the absence of any carry-over effect was checked, since for all analytes, the blank samples injected after the higher level of calibration curve had no relevant signal.

### Application to real specimens

Fentanyl, like many other drugs, can cross the placental barrier and thus reach the fetus, even when administered in epidural anesthesia [27]. Moreover, identification of several drugs has been reported in meconium after intake during labor [28–30]. Therefore, 30 meconium specimens from newborns whose mothers were administered this drug through epidural anesthesia were analyzed to verify the possibility to detect fentanyl and/or its main metabolites. These specimens were from cases in which fentanyl had been administered as epidural anesthesia at the time of delivery (*n* = 27) or cases in which the maternal hair was positive to other drug of abuse (*n* = 3) tested after delivery.

Four meconium specimens tested positive for fentanyl (range 440–750 ng/g) and two specimens tested positive to acetylfentanyl (range 190–1400 ng/g). Three of the fentanyl-positive meconium samples (case 10, 13, and 26; at the concentration of 520 ng/g, 450 ng/g, and 750 ng/g, respectively) were cases in which fentanyl was administered as epidural anesthesia. Moreover, in case 13, the maternal hair also tested positive for fentanyl (5.0, 5.7, and 4.9 pg/mg for the first, second, and third trimesters, respectively) by using a validated method [22]. But the concentrations in hair were very low and fentanyl was also detected in the last wash, probably due to a contamination during labor. Finally, in the fourth case which tested positive (case 30; at the concentration of 440 ng/g), fentanyl was not administered as epidural anesthesia or detected in maternal hair, an unauthorized intake of fentanyl has likely occurred. Furthermore, the maternal hair tested positive for MDMA in the first trimester of pregnancy (162.2 pg/mg). Quite remarkably, norfentanyl was never detected in meconium, albeit the relatively high concentrations of fentanyl and the low LOD for norfentanyl (0.5 ng/g). This finding suggests that fentanyl is poorly metabolized or barely adsorbed by newborns, opening two different scenarios about the effects of fentanyl administrations to infants which would deserve further evaluations.

**Fig. 1:**
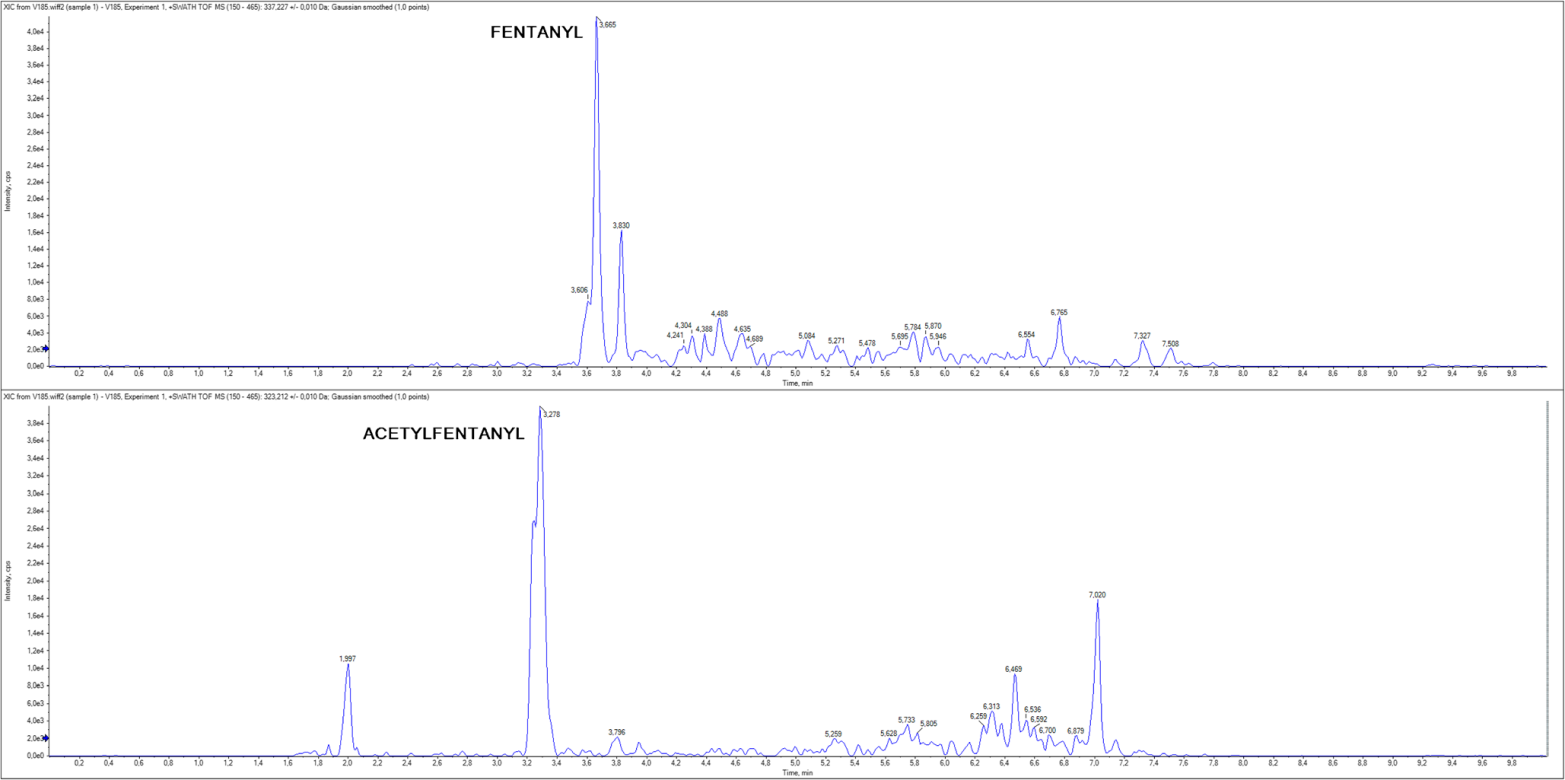
Chromatogram from a real sample positive to fentanyl and acetylfentanyl

Figure 1 shows the chromatogram from a real positive sample. Table 4 shows the positive meconium specimens along with the information about the epidural anesthesia received by the mother and results in maternal hair.

**Table 4:** Positive meconium specimens (semiquantitative results), information about the epidural anesthesia, and results in maternal hair

Specimen	Compound	Concentration (ng/g)	Epidural anesthesia (fentanyl)	Fentanyl in maternal hair (pg/mg)
10	Fentanyl Acetylfentanyl	520 1400	Yes	
13	Fentanyl Acetylfentanyl	450 190	Yes	1° trim: 5.0 2° trim: 5.7 3° trim: 4.9
26	Fentanyl	750	Yes	
30	Fentanyl	440	No	

Empty boxes represent negative results

*Trim* trimester

## Conclusions

A method that allows the simultaneous determination of 137 new psychoactive substances (including synthetic cathinones, hallucinogens, synthetic cannabinoids, fentanyl analogs, and other synthetic opioids) in meconium was developed and validated for semi-quantitative purpose. This was the first attempt to use new technologies such as QTOF mass spectrometry for a broad-spectrum drug screening in meconium. In terms of analytical performances, the method proved fit for its purpose. In particular, the very low LODs seem adequate to detect the presence of NPS after the ingestion of active doses from the mother. Unfortunately, the limited number of real samples, and especially the lack of real samples positive to targeted compounds other than fentanyl, prevent us to confirm the former statement. A further limitation of this analytical method is the cumbersome sample preparation prior the analysis (homogenization and SPE). However, meconium is a very complex biological matrix, so its analysis using simpler processes such as “dilute and shoot” is not possible. On the other hand, HRMS screening methods can become of particular interest in the modern drug market dominated by NPS. In fact, the UHPLC-QTOF equipment provides high mass accuracy and accurate isotopic patterns which in turn allows confidence for the identification of NPS. Furthermore, newly discovered NPS can be added to the panel of target analytes to look for the presence of these new substance without adjusting the extraction method, which is very promising with the continuously changing drug market of NPS. In the future, only the routinary application of drug screening in meconium will allow to better understand the actual prevalence of NPS consumption among pregnant women.
